# Prevalence of Knee Pain and Its Relation to Depression, Anxiety, and Health-Related Quality of Life Among Maintenance Hemodialysis Patients

**DOI:** 10.3390/jcm14020368

**Published:** 2025-01-09

**Authors:** Samar Tharwat, Eman Nagy, Abdelrahman Mohammed Elsayed, Karem Mohamed Salem, Ahmed M. Salah, Sherin Zohdy Mohamed, Mohammed Kamal Nassar

**Affiliations:** 1 Rheumatology & Immunology Unit, Department of Internal Medicine, Faculty of Medicine, Mansoura University, Mansoura 35516, Egypt; 2 Department of Internal Medicine, Faculty of Medicine, Horus University, New Damietta 34517, Egypt; shzohdy@horus.edu.eg (S.Z.M.); m_kamal@mans.edu.eg (M.K.N.); 3 Mansoura Nephrology & Dialysis Unit (MNDU), Department of Internal Medicine, Faculty of Medicine, Mansoura University, Mansoura 35516, Egypt; emannagy@mans.edu.eg; 4 Faculty of Medicine, Mansoura University, Mansoura 35516, Egypt; abdorezk588@gmail.com; 5 Nephrology & Dialysis Unit, Department of Internal Medicine, Faculty of Medicine, Fayoum University, Fayoum 63515, Egypt; kariemsalem@gmail.com; 6 Nephrology Unit, Department of Internal Medicine, Faculty of Medicine, Zagazig University, Zagazig 44519, Egypt; ahmedsalah_400@yahoo.com

**Keywords:** knee pain, WOMAC, HRQoL, HADS, hemodialysis, ESRD

## Abstract

**Background/Objectives**: Knee pain in hemodialysis (HD) patients might affect health-related quality of life (HRQoL) and may be related to anxiety and depressive symptoms. The aim of this study was to assess the prevalence of knee pain in chronic HD patients and to determine its relationship with anxiety, depression, and HRQoL, **Methods**: This multicenter cross-sectional study was carried out on chronic HD patients. Sociodemographic, clinical, and therapeutic data were collected. The Knee Pain Screening Tool (KNEST) was used to screen for knee pain. Patients with knee pain were instructed to complete the visual analog scale (VAS) for pain and the Western Ontario and McMaster Universities Arthritis Index (WOMAC). The patients also completed an Arabic-language version of the Hospital Anxiety and Depression Scale (HADS) and the Kidney Disease Quality of Life-36 (KDQOL-36™) questionnaire. **Results**: This study included 271 chronic HD patients; the median age was 51 (IQR 21) years, and most of them were males (59%). Of them, 158 had knee pain. Those with knee pain were more likely to have anxiety compared to those without (*p* = 0.002) and significantly lower scores on the symptom/problem (*p* = 0.03) and burden of kidney disease domains (*p* = 0.047) and the physical health (*p* < 0.001) and mental health components (*p* = 0.001). Furthermore, those with moderate to severe knee pain were more likely to experience anxiety (*p* = 0.001) and depression (*p* = 0.005) and have a lower physical health composite (PHC) than those with mild knee pain (*p* = 0.046). **Conclusions**: HD patients have a significant prevalence of knee pain that is usually associated with anxiety and leads to worse HRQoL than those without knee pain.

## 1. Introduction

The global burden of chronic kidney disease (CKD) has increased significantly over the past three decades [1]. Hemodialysis (HD) accounts for 89% of all kidney failure (KF) treatments worldwide [2]. Despite the advancements in HD, it can have detrimental impacts on patients’ general health and well-being, as well as on their physical performance [3]. Musculoskeletal problems, such as dialysis-associated arthropathy and chronic kidney disease–mineral and bone disorders (CKD–MBDs), continue to be a significant issue for long-term HD patients [4]. Almost all HD patients experience at least one musculoskeletal problem [5]. Among these individuals, muscular cramps, myalgias, and arthralgias are the most prominent musculoskeletal diseases [6].

Patients’ health-related quality of life (HRQoL) can be negatively impacted by persistent musculoskeletal problems, which interfere with sleep, memory, and social and/or physical activities [6,7]. Additionally, musculoskeletal symptoms may be associated with mental disorders like anxiety and depression in maintenance HD patients. Chronic musculoskeletal pain and depression typically coexist [8].

Knee pain is a common musculoskeletal complaint among HD patients; a recent study of 200 HD patients revealed that knee pain is the most prevalent musculoskeletal symptom, affecting 51.5% of participants [5]. Individuals on chronic HD commonly experience severe knee joint OA and osteonecrosis as a result of both the natural aging process and the accumulation of β2-microglobulin [9].

HRQoL is a subjective and multidimensional concept comprising physical, psychological, and social functioning in relation to a health condition or therapy [10]. While the impact of knee pain on HRQoL has been explored in the literature [11,12], there is still a lack of knowledge regarding its impact on HRQoL in HD patients. Moreover, several studies have highlighted the need for identifying and managing psychological factors associated with physical function and knee pain throughout rehabilitation [13,14]. Although relevant, there is insufficient data available on anxiety and depression as potential predictors of knee pain in patients with chronic HD. Binik YM and coauthors found that self-reported depression was associated positively with pain in HD patients [15]. Kidney diseases are also strongly linked with the exacerbation of knee pain and correlate with the progression of functional disability [16]. Many individuals undergoing HD require total knee arthroplasty due to severe knee joint arthritis [9]. However, no study has investigated knee pain specifically in HD patients and its relation to anxiety and depression. So, we conducted this study to evaluate the prevalence of knee pain and its correlation with anxiety, depression, and HRQoL in chronic HD patients.

## 2. Materials and Methods

### 2.1. Study Design and Settings

This was a multicenter cross-sectional study of 271 KF patients on chronic HD recruited from multiple HD centers in multiple Egyptian governorates: Dakahlia, Cairo, Fayoum, and Gharbia. The study was conducted during the period between June and December 2021. The inclusion criteria were individuals over the age of 18 who had been on HD for more than six months. Patients with cognitive impairment, terminal malignancy, advanced organ dysfunction, autoimmune or rheumatic disease, or hearing or visual abnormalities were excluded from the study.

The sample size calculation was conducted based on G*Power software (Manufacturer: Heinrich-Heine-University Düsseldorf, Düsseldorf, Germany, Version: 3.1.9.3). The outcome of interest was the prevalence of knee pain among HD patients, which was 52% [5], with an effect size of 0.1, an alpha error of 0.05, and a power of study of 0.9. So, the sample size was found to be 269 subjects, which is the minimum sample size required. The participants’ number exceeded the calculated sample size. As a result, the final number of participants included was 271. We distributed the total sample proportionally among HD units in Dakahlia and Gharbia and collected it using a systematic random sampling method. Prior to their enrollment in the study, all patients provided written informed consent. The study was approved by the Mansoura Faculty of Medicine Institutional Research Board (approval number: R.21.10.1465.R1).

### 2.2. Sociodemographic Data and Clinical Characteristics

The patients’ records were examined for sociodemographic information, including age, sex, marital status, residence, level of education, smoking status, and socioeconomic status. In addition, relevant clinical data, such as the duration of HD, the existence of diabetes, hypertension, and/or other comorbidities, and medication history, were documented.

### 2.3. Knee Pain Screening Tool (KNEST) and Visual Analog Scale (VAS)

The KNEST is a brief knee pain screening tool. It consists of six questions, as follows: Previous knee injury? Knee pain last year? Laterality of knee pain? Chronicity? General practitioner (GP) consultation last year? Other healthcare uses? [17,18]. The KNEST is a reliable and valid composite tool for studying population needs and treatment outcomes for people with knee pain [19].

Patients with knee pain were instructed to complete the visual analog scale (VAS) for pain, in which the line represented a spectrum ranging from no pain to the worst possible pain and was graded as follows: no pain (0 points), mild pain (1–3 points), moderate pain (4–6 points), and severe pain (7–10 points) [20].

### 2.4. The Western Ontario and McMaster Universities Arthritis Index (WOMAC)

The WOMAC is a widely used, self-administered tool used to assess pain, joint stiffness, and the degree of difficulty in performing daily activities in patients with knee or hip pain. It consists of the following 24 questions: five questions for pain, two questions for stiffness, and 17 questions about the level of difficulty in doing daily life activities. The questions are scored using a five-point Likert scale with degrees of response for each topic, representing varying intensities: (0) none, (1) mild, (2) moderate, (3) severe, and (4) extreme. In this study, knee pain was evaluated using the Arabic version of the WOMAC. The Arabic version of the WOMAC index is a reliable and valid tool for assessing knee osteoarthritis intensity, with measurement features that align with those of the original version [21]. On a visual analog scale, the severity of knee pain was classified as follows: mild (1–3), moderate (4–6), and severe (7–10) [22].Cronbach’s Alpha for the WOMAC subscales was 0.92 for pain, 0.90 for stiffness, and 0.98 for physical function [23].

### 2.5. Hospital Anxiety and Depression Scale (HADS)

The patients completed an Arabic-language version of the HADS questionnaire, which is a valid and reliable tool to assess mood states [24]. Cronbach’s alpha was found to be 0.78–0.93 for HAD-A and 0.82–0.90 for HAD-D [25]. The HADS questionnaire employs two distinct subscales to measure anxiety and depression. Each question is scored on a four-point Likert scale (from 0 to 3). Patients with a score of 7 or less are regarded as normal in terms of depression and anxiety; those with a score of 8–10 are thought to be borderline instances of depression and anxiety; and those with a score of 11–21 are considered abnormal in terms of depression and anxiety (cases) [24]. The Arabic HADS has good reliability and internal consistency, which warrants its use in screening for anxiety and depression among Arabic-speaking HD patients [26].

### 2.6. The Kidney Disease Quality of Life-36 (KDQOL-36)

We assessed the HRQoL of the studied patients using KDQOL-36, a reliable and valid tool. Cronbach’s alpha for the scale was 0.92 (*p* < 0.001), and the alphas for the five subscales ranged from 0.76 to 0.92 (*p* < 0.001) [27]. The original version included the Medical Outcomes Study 36 as a generic chronic illness core, as well as items specific to kidney disease patients. It had 36 questions, with questions 1–12, 1–12, 13–16, 17–28, and 29–36 obtaining the mental health composite (MHC) and physical health composite (PHC), burden of renal disease, symptom/problem list, and effect of kidney disease components of HRQoL, respectively. These five KDQOL-36 components have average values ranging from 0–100, with higher scores indicating better HRQoL [28].

### 2.7. Blood Sampling and Laboratory Tests

Blood samples were obtained from the arteriovenous fistula just before the first HD session of the week. An automated analyzer was used to perform laboratory tests [29] on the same day of blood sampling. These tests included hemoglobin (HB) level, calcium, phosphorus, and albumin, as well as transferrin saturation (TSAT) (using Cobas c311 Roche diagnostics), serum ferritin, and intact parathyroid hormone (iPTH) (using Cobas e411 Roche diagnostics).

### 2.8. Statistical Analysis

The collected data were coded, processed, and analyzed using the Statistical Package for Social Science (SPSS) version 25 for Windows on personal computers. Categorical data were described as percentages and numbers, while continuous data were described as means [±standard deviation (SD)] for normally distributed variables or medians (IQR) for non-normally distributed variables, as suitable. To assess the normality of the distribution of the variables, the Kolmogorov–Smirnov test was used. For comparing two groups, Student’s *t*-test was used for normally distributed variables, while the Mann–Whitney test was used for non-normally distributed variables. The Chi-square test was used for comparing categorical variables. For comparing three groups, the one-way ANOVA test was used for normally distributed variables, while the Kruskal–Wallis test was used for non-normally distributed variables. The level of significance was considered 5% (*p* ≤ 0.05). The effect size was calculated for two groups as Cohen’s d and for more than two groups as Eta squared.

## 3. Results

### 3.1. Sociodemographic Data and Clinical Characteristics

In this study, 271 KF patients on chronic HD were recruited from multiple HD facilities in two Egyptian governorates. The median (IQR) age was 51 years (21). The majority of patients were male (59%). Approximately three-quarters were married (72.7%). According to the existence of knee pain, 113 patients were classified as not having knee pain, whereas 158 were classified as having knee pain. The characteristics of HD patients with and without knee pain are depicted in Table 1. There were no statistically significant differences between the non-knee pain and knee pain groups regarding marital status, place of residence, or smoking status. Those experiencing knee pain were significantly older (*p* = 0.013).

Comparing the clinical features and laboratory data of the two groups, we discovered that patients with knee pain had a longer duration since beginning HD (*p* = 0.004) and a lower HB level (*p* = 0.02), as shown in Table 2.

**Figure 1:**
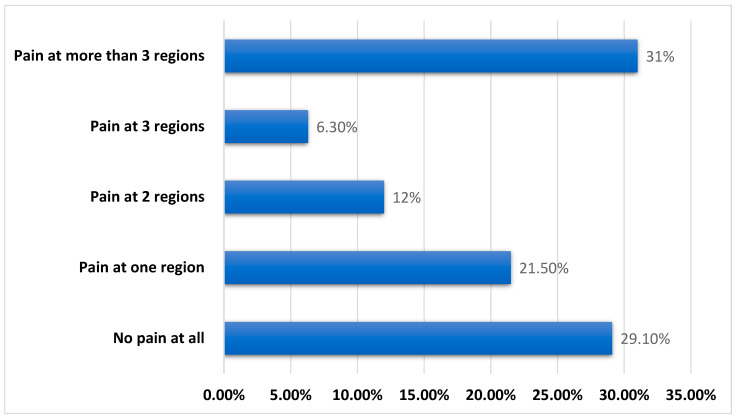
Associated musculoskeletal pain among hemodialysis patients with knee pain (*n* = 158).

Regarding other associated musculoskeletal pain in the knee pain group, 29% reported no pain in any other region, 22% described at least one region of pain, 12% described at least two regions, and 6% described at least three regions, as depicted in Figure 1. Approximately one-third of the knee pain group (33%) reported pain in more than three regions.

In the knee pain group, 29% reported no other concomitant musculoskeletal pain, 22% described at least one site of pain, 12% identified at least two regions, 6% mentioned at least three places, and 33% of the knee pain group experienced pain in more than three locations, as shown in Figure 1.

### 3.2. Knee Pain Screening Tool (KNEST)

**Figure 2:**
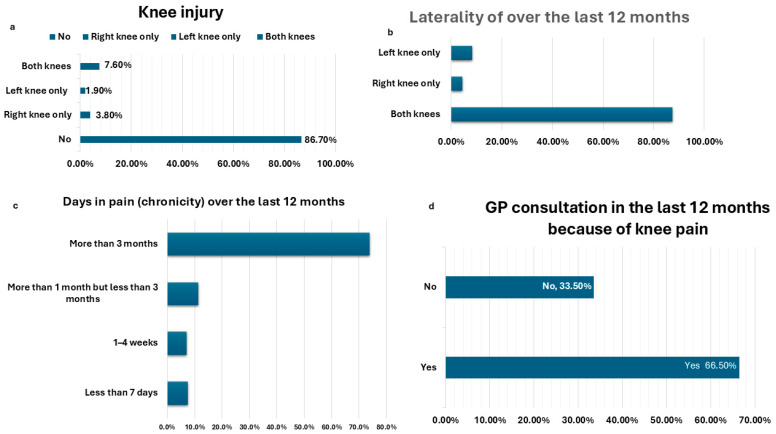
Knee Pain Screening Tool (KNEST) among hemodialysis patients with knee pain (*n* = 158); (**a**) history of knee injury, (**b**) laterality of knee pain, (**c**) days in pain (chronicity), (**d**) GP consultation in the last 12 months because of knee pain.

In patients with knee pain, the knee pain screening instrument was administered. Eighty-seven percent had no history of knee injuries, whereas 4% had a right knee injury, 2% had a left knee injury, and 7% had injuries to both knees. Almost all of them (88%) reported bilateral knee pain. Seventy-four percent experienced knee pain for more than three months within the past year, while just 7.6% reported pain for less than seven days. Figure 2 depicts that 66% of respondents visited their primary care physician over this issue within the past year.

### 3.3. Hospital Anxiety and Depression Scale (HADS) and the Kidney Disease Quality of Life-36 (KDQOL-36)

After calculating the scores of the responses to the questions about anxiety, the participants were categorized as normal (22.1%), borderline abnormal (26.9%), and abnormal (50.9%). In the same context, after calculating the scores for the responses to the questions about depression, the participants were categorized as normal (15.5%), borderline abnormal (29.9%), and abnormal (54.6%). The participants with scores classified as abnormal were considered to have either anxiety and/or depression, depending upon the section of the questionnaire.

The relationship between anxiety and depression scores and knee pain is illustrated in Table 3. This study used HRQoL domain scores based on whether knee symptoms were present or absent. Patients with knee pain had significantly lower symptom/problem (*p* = 0.003), burden of kidney disease (*p* = 0.047), physical health (*p* < 0.001), and mental health (*p* = 0.001) component scores (Table 3).

### 3.4. The Effect of Pain Severity Among Patients with Knee Pain

According to the VAS, 12 (7.6%) of those with knee pain had mild pain, 95 (60.1%) had moderate pain, and 51 (32.3%) had severe pain. Those in moderate to severe pain exhibited statistically significantly higher WOMAC scores, including the pain, stiffness, and physical domains. Furthermore, those with moderate to severe knee pain were more likely to experience anxiety (*p* = 0.001), depression (*p* = 0.005), and a lower PHC (*p* = 0.046) than those with mild knee pain, as shown in Table 4.

According to the VAS, 12.7% of those with knee pain experienced mild pain, 60.1% experienced moderate pain, and 32.3% experienced severe pain. Those with moderate to severe pain had significantly higher WOMAC scores for the pain, stiffness, and physical domains. In addition, as shown in Table 4, those with moderate to severe knee pain were more likely to suffer anxiety (*p* = 0.001) and depression (*p* = 0.005) and have a lower PHC (*p* = 0.046) than those with mild knee pain.

## 4. Discussion

This is the first thorough examination of knee pain in HD patients using the KNEST and the HADS questionnaire. This study examined the association between knee pain and anxiety, depression, and HRQoL in patients with RF who had been on chronic HD for more than six months. This study found that HD patients had an elevated prevalence of knee pain, which was associated with anxiety and a decline in HRQoL. It also demonstrated that HD patients who complained of knee pain may compromise their physical and mental health. Patients with HD experience a plethora of musculoskeletal complaints [5]. The prevalence of knee pain, as reported by 58% of the patients, is one of the most significant findings of the current study. Those who reported knee pain were somewhat older. This was predictable, given that knee pain is a common physical complaint among older people [30]. Increased joint pain and decreased joint range of motion are common complaints among older people and are frequently attributable to OA [31,32]. Muscle strength is vital for physical function, and levels vary with age, sex, and body composition. Muscle strength is changed throughout life by physical activity and training techniques [33]. Knee extensor muscle weakness has been associated with higher knee pain in those with or at risk for knee OA [34]. This could explain why the participants with knee pain in our study reported less physical activity.

In this study, patients with knee pain had a longer duration since beginning HD and a lower HB level. This is consistent with previous research that demonstrated a significant association between knee pain and dialysis vintage [4,35,36]. Musculoskeletal complaints become more prevalent as the number of years on dialysis increases. Multifactorial mechanisms underlie persistent musculoskeletal pain in HD patients [37].

Seventy-one percent of the 158 HD patients with knee pain reported musculoskeletal pain in other regions; 22% reported pain in at least one region, 12% in at least two regions, and 6% in at least three regions. This is consistent with a previous study of 200 patients with chronic HD that found that 90% of them had musculoskeletal pain [5].

In this study, we found that chronic HD patients with knee pain were more significantly likely to have anxiety than those without knee pain. Anxiety increases sensory pain thresholds both at the site of the disease and in distant locations, as well as the self-reporting of pain by an individual [38]. Anxiety is a future-oriented affective disorder with a hazy treatment focus. Anxiety contributes to the fear aversion model in the musculoskeletal system and is associated with preventative behaviors. This avoidance model may eventually lead to inactivity and disability [39]. Negative effects, such as anxiety and depression, are associated with decreased pain thresholds in healthy individuals [40], and this is exacerbated in chronic pain states, such as musculoskeletal disorders [41].

This study utilized the HRQoL domain scores based on whether knee symptoms were present or absent. Patients with knee pain had significantly lower scores on the symptom/problem and burden of kidney disease domains, as well as the physical health and mental health components. Patients with ESRD are well-known to have a diminished HRQoL [42]. Pain is associated with negative outcomes in patients with chronic HD, including altered sleep and depression, as well as a decline in HRQoL [43,44]. In this population, pain is one of the most common symptoms [45]. In these patients, knee pain particularly restricts their mobility, which is already compromised by other factors, such as prolonged periods of sitting on HD machines and sarcopenia. Consequently, their HRQoL decreases. This decline in HRQoL can lead to increased psychological distress, further exacerbating patients’ overall well-being. Addressing these multifaceted issues is crucial in developing comprehensive care plans that improve both mobility and HRQoL for these patients.

In a study conducted in Sweden on a random sample of 1300 individuals with knee pain for four weeks and 650 individuals without knee pain, the participants with knee pain reported lower HRQoL scores than the participants without knee pain [46]. In another cross-sectional study involving data from the OA initiative project and 1252 older adults (≥65 years), bilateral knee pain was significantly associated with lower physical and mental HRQoL after controlling for all confounding variables [47]. In addition, Bindawas and coworkers gathered data from OA initiative public-use data sets and assessed HRQoL in 2481 participants (aged 45–79 years at baseline). Patients with frequent bilateral knee pain reported a lower HRQoL than those with frequent unilateral knee pain or no knee pain [48].

In addition, those with moderate to severe knee pain were more prone to anxiety and depression and had a lower PHC than those with mild knee pain. In healthy individuals, a lower pain threshold is associated with negative effects, including anxiety and depression [40]. Anxiety and depression have direct effects on both reported current pain and reported pain one week later [49]. In human imaging studies, the activity of the anterior cingulate cortex (ACC) during pain anticipation has been related to the intensity of anxiety–pain interactions [50].

This study has some limitations. Due to the cross-sectional design, all findings can only be considered associations; no conclusions can be drawn regarding causality. Second, all data were self-reported by the study participants, and no clinical diagnoses were made. Even though the desired condition for examining the association between knee pain and HRQoL is to compare them to a community control group, the absence of a control group and potential confounders is one of our limitations in this study. It would be better to determine the cause of the knee pain because it would add a lot to the study. By understanding the underlying factors contributing to pain, researchers can develop targeted therapies that not only alleviate symptoms but also address the root issues. Despite these limitations, this study highlighted the high prevalence of knee pain in HD patients, its impact on HRQoL, and its association with anxiety and depression symptoms. Future research could address confounding factors by incorporating matched control groups, such as HD patients without knee pain or non-hemodialysis individuals with comparable demographic profiles, to better study the effects of knee pain and its psychological impact. Longitudinal studies are also recommended to explore causal relationships between knee pain, anxiety, depression, and HRQoL. Additionally, controlling for potential confounders, such as comorbid conditions, medication use, and lifestyle factors through stratified analysis or regression models could provide more nuanced insights. Employing objective clinical assessments alongside self-reported measures may further enhance the reliability of findings.

## 5. Conclusions

In conclusion, HD patients have a high prevalence of knee pain, which is typically associated with anxiety. Furthermore, patients with knee pain have significantly lower HRQoL than those without. Therefore, early intervention is necessary, including physical therapy, strengthening exercises, and educational programs to improve joint health. These strategies aim to reduce discomfort, enhance mobility, and prevent further deterioration of knee function. This may mitigate the medical, social, and economic consequences of knee pain for these individuals.

## Figures and Tables

**Table 1:** Sociodemographic data of studied hemodialysis patients (*n* = 271).

Variables Mean ± SD, Median (IQR), *n* (%)	Patients Without Knee Pain (*n* = 113)	Patients with Knee Pain (*n* = 158)	*p* Value	Effect Size #	95% CI of Difference
Age (years)	48 (20.50)	53 (22)	**0.013** *	0.357	−7.50, −1.1
Sex					
Male	81 (71.7)	79 (50)	**<0.001** *	-	-
Female	32 (28.3)	79 (50)			
Marital status					
Single/divorced/widowed	27 (23.9)	47 (29.7)	0.286	-	-
Married	86 (76.1)	111 (70.3)			
Residence					
Rural	66 (58.4)	77 (48.7)	0.116	-	-
Urban	47 (41.6)	81 (51.3)			
Education					
Not educated	21 (18.6)	52 (32.9)			
Low School	13 (11.5)	18 (11.4)			
Middle School	12 (10.6)	12 (7.6)	0.136	-	-
High School	45 (39.8)	57 (36.1)			
College degree	20 (17.7)	17 (10.8)			
Post-graduate	2 (1.8)	2 (1.3)			
Socioeconomic status					
Low	50 (44.2)	73 (46.2)			
Average	61 (54)	82 (51.9)	0.944	-	-
High	2(1.8)	3 (1.9)			
Smoking					
Never	59 (52.2)	87 (55.1)			
Former smoker	35 (31)	47 (29.7)	0.886	-	-
Current smoker	19 (16.8)	24 (15.2)			

* statistically significant, # Effect size assessed by Cohen’s d. Data are expressed as number (percentage) except age as median (min–max). Numbers in bold indicate that the *p* value is less than 0.05

**Table 2:** Clinical characteristics and therapeutic and laboratory data of studied hemodialysis patients (*n* = 271).

Variables	Patients Without Knee Pain (*n* = 113)	Patients with Knee Pain (*n* = 158)	*p* Value	Effect Size #	95% CI of Difference
Duration of hemodialysis (years)	4 (6.00)	7 (8.00)	**0.004** *	0.30	−2.7, −0.29
Associated comorbidities					
Diabetes	16 (14.2)	23 (14.6)	0.927		
Hypertension	71 (62.8)	112 (70.9)	0.163		
Chronic respiratory disease	1 (0.9)	3 (1.9)	0.495	-	-
Psychiatric disorder	0	9 (5.7)	-		
Ischemic heart disease	10 (8.8)	6 (3.8)	0.082		
Therapeutic data					
Erythropoietin	67 (59.3)	88 (55.7)	0.555		
Calcium supplementations	102 (90.3)	133 (84.2)	0.145		
Iron supplementations	93 (82.3)	125 (79.1)	0.514	-	-
Antihypertensive drugs	67 (59.3)	121 (76.6)	**0.002**		
Antidiabetic drugs	25 (22.1)	34 (21.5)	0.905		
Laboratory data					
Blood hemoglobin (gm/dL)	10.67 ± 1.3	10.26 ± 1.3	**0.02** *	0.32	0.063, 0.755
Serum ferritin (ng/mL)	265.7 (414.15)	281.3 (237.38)	0.811	0.02	−154.68, 144.59
TSAT (%)	20.5 (8.75)	20.5 (12.25)	0.895	0.08	−4.87, 3.69
Serum calcium (mg/dL)	8.4 (0.88)	8.6 (0.58)	0.790	0.02	−0.22, 0.276
Serum phosphorus (mg/dL)	4.6 (2.85)	4.5 (1.85)	0.254	0.23	−0.045, 0.66
iPTH (pg/mL)	405.5 (507)	371 (564)	0.666	0.07	−233.72, 166.33
Serum albumin (gm/dL)	3.95 (0.20)	4 (0.40)	0.270	0.25	−0.19, 0.74

iPTH: intact parathyroid hormone, TSAT: Transferrin saturation, * statistically significant; # Effect size assessed by Cohen’s d; Data are expressed as median (min–max) except blood hemoglobin as mean ± SD and associated comorbidities and therapeutic data as number (percentage). Numbers in bold indicate that the *p* value is less than 0.05

**Table 3:** Anxiety and depression scores and health-related quality of life of studied hemodialysis patients with and without knee pain (*n* = 271).

Variables Median (IQR), *n* (%)	Patients Without Knee Pain (*n* = 113)	Patients with Knee Pain (*n* = 158)	*p* Value	Effect Size #	95% CI of Difference
Anxiety					
Normal	37 (32.7)	23 (14.6)			
Borderline abnormal	26 (23)	47 (29.7)	**0.002** *		
Abnormal	50 (44.2)	88 (55.7)			
Depression					
Normal	24 (21.2)	18 (11.4)			
Borderline abnormal	30 (26.5)	51 (32.3)	0.080		
Abnormal	59 (52.2)	89 (56.3)			
Health-related quality of life					
Symptom/problem list	75 (20.83)	66.67 (27.09)	**0.003** *	0.341	1.93–11.6
Effect of kidney disease	68.75 (25.00)	65.63 (28.12)	0.501	0.101	−2.93, 7.13
Burden of kidney disease	18.75 (31.25)	12.5 (25.00)	**0.047** *	0.195	−0.93, 8.61
PHC	34.69 (9.57)	30.65 (8.83)	**<0.001** *	0.503	1.80–5.11
MHC	37.59 (10.80)	35.6 (7.98)	**0.001** *	1.04	1.74–5.48

MHC: mental health composite, PHC: physical health composite, * statistically significant; # Effect size assessed by Cohen’s d; Anxiety and depression are described as number (percentage) and all other parameters as median (range). Numbers in bold indicate that the *p* value is less than 0.05

**Table 4:** WOMAC, anxiety, and depression scores and health-related quality of life of the studied hemodialysis patients according to the severity of knee pain by VAS.

Variables Median (IQR), *n* (%)	Patients with Knee Pain (*n* = 158)				Effect Size #	95% CI of Difference
	Mild (*n* = 12)	Moderate (*n* = 95)	Severe (*n* = 51)	*p*		
WOMAC	34.4 (27.60) ^1,2^	55.2 (19.79) ^1^	53.1 (29.17) ^2^	**0.009** *	**0.202**	CI_1_ = −36.69, −12.11 CI_2_ = −42.63, −17.01 CI_3_ = −2.37, 13.22
Pain	5 (4.00) ^1,2^	9 (5.00) ^1,3^	10 (6.00) ^2,3^	**<0.001** *	**0.23**	CI_1_ = −7.29, −3.09 CI_2_ = −9.62, −5.13 CI_3_ = 0.786, 3.58
Stiffness	2 (3.50) ^1,2^	4 (3.00) ^1,3^	4 (4.00) ^2,3^	**<0.001** *	**0.16**	CI_1_ = −2.57, −0.672 CI_2_ = −3.66, −1.64 CI_3_ = 0.391, 1.67
Physical	25 (27.00) ^1,2^	39 (16.00) ^1^	39 (26.00) ^2^	**0.009** *	**0.19**	CI_1_ = −24.81, −7.77 CI_2_ = −29.45, −11.65 CI_3_ = −1.0, 9.56
Anxiety						
Normal	5 (41.6) ^1^	5 (5.3) ^1,2^	13 (25.5) ^2^			
Borderline abnormal	5 (41.6)	38 (40)	4 (7.8)	**<0.001** *	-	-
Abnormal	2 (16.8)	52 (54.7)	34 (66.7)			
Depression						
Normal	4 (33.3) ^1^	5 (5.3) ^1,2^	11 (21.6) ^2^			
Borderline abnormal	1 (8.3)	32 (33.6)	15 (29.4)	**0.005** *	-	-
Abnormal	7 (58.4)	58 (61.1)	25 (49)			
Health-related quality of life						
Symptom/problem list	83.3 (35.41)	64.6 (23.96)	66.7 (30.21)	0.143	**0.02**	CI_1_ = −1.81, 19.55 CI_2_ = 3.26, 25.91 CI_3_ = −12.57, 1.14
Effect of kidney disease	68.7 (28.13)	65.6 (31.25)	62.5 (37.50)	0.582	0.012	CI_1_ = −6.21, 15.8 CI_2_ = −7.27, 16.17 CI_3_ = −6.71, 7.48
Burden of kidney disease	18.75 (56.25)	12.5 (25.00)	6.25 (25.00)	0.648	0.05	CI_1_ = 0.491, 21.30 CI_2_ = −0.356, 21.71 CI_3_ = −6.46, 6.90
PHC	35.2 (11.08) ^1,2^	30.2 (7.31) ^1^	29.2 (10.58) ^2^	**0.046** *	0.04	CI_1_ = 1.466, 7.96 CI_2_ = 2.06, 8.95 CI_3_ = −2.87, 1.29
MHC	43.9 (9.35)	35.6 (7.00)	35.4 (11.19)	0.058	0.04	CI_1_ = 0.523, 8.23 CI_2_ = −0.989, 7.18 CI_3_ = −1.19, 3.75

WOMAC: Western Ontario and McMaster Universities Arthritis Index, * statistically significant; The similar superscripted numbers in the same row denote significant differences between severity degree. # Effect size assessed by Eta squared. CI_1_: Calculated for mean difference between mild versus moderate cases, CI_2_: Calculated for mean difference between mild versus severe cases, CI_3_: Calculated for mean difference between severe versus moderate cases. Anxiety and depression are described as number (percentage) and all other parameters as median (range).

## Data Availability

The data sets used and/or analyzed during the current study are available from the corresponding author on reasonable request.

## References

[1] Bikbov B., Purcell C.A., Levey A.S., Smith M., Abdoli A., Abebe M., Adebayo O.M., Afarideh M., Agarwal S.K., Agudelo-Botero M. (2020). Global, regional, and national burden of chronic kidney disease, 1990–2017: A systematic analysis for the Global Burden of Disease Study 2017. Lancet.

[2] Liyanage T., Ninomiya T., Jha V., Neal B., Patrice H.M., Okpechi I., Zhao M.-h., Lv J., Garg A.X., Knight J. (2015). Worldwide access to treatment for end-stage kidney disease: A systematic review. Lancet.

[3] Valderrábano F., Jofre R., López-Gómez J.M. (2001). Quality of life in end-stage renal disease patients. Am. J. Kidney Dis..

[4] Hage S., Hage V., El-Khoury N., Azar H., Chelala D., Ziadé N. (2020). Musculoskeletal disorders in hemodialysis patients: Different disease clustering according to age and dialysis vintage. Clin. Rheumatol..

[5] Ezzat S., Tharwat S., Abdelsalam S., Eltoraby E.E. (2020). Musculoskeletal Symptoms in Hemodialysis Patients and their Effect on Health-Related Quality of Life. Blood Purif..

[6] Fidan F., Alkan B.M., Tosun A., Altunoğlu A., Ardıçoğlu Ö. (2016). Quality of life and correlation with musculoskeletal problems, hand disability and depression in patients with hemodialysis. Int. J. Rheum. Dis..

[7] Santoro D., Satta E., Messina S., Costantino G., Savica V., Bellinghieri G. (2013). Pain in end-stage renal disease: A frequent and neglected clinical problem. Clin. Nephrol..

[8] Poleshuck E.L., Bair M.J., Kroenke K., Damush T.M., Tu W., Wu J., Krebs E.E., Giles D.E. (2009). Psychosocial stress and anxiety in musculoskeletal pain patients with and without depression. Gen. Hosp. Psychiatry.

[9] Kii S., Sonohata M., Hashimoto A., Nakashima T., Kawaguchi A., Matsumura Y., Shimazaki T., Nagamine S., Mawatari M. (2021). Mid-term clinical outcomes and complications of primary total knee arthroplasty in hemodialysis patients: A retrospective comparative cohort study. BMC Musculoskelet. Disord..

[10] Revicki D.A. (1989). Health-related quality of life in the evaluation of medical therapy for chronic illness. J. Fam. Pract..

[11] Hill C.L., Parsons J., Taylor A., Leach G. (1999). Health related quality of life in a population sample with arthritis. J. Rheumatol..

[12] Muraki S., Akune T., Oka H., En-yo Y., Yoshida M., Saika A., Suzuki T., Yoshida H., Ishibashi H., Tokimura F. (2010). Association of radiographic and symptomatic knee osteoarthritis with health-related quality of life in a population-based cohort study in Japan: The ROAD study. Osteoarthr. Cartil..

[13] Phyomaung P.P., Dubowitz J., Cicuttini F.M., Fernando S., Wluka A.E., Raaijmaakers P., Wang Y., Urquhart D.M. (2014). Are depression, anxiety and poor mental health risk factors for knee pain? A systematic review. BMC Musculoskelet. Disord..

[14] de Rooij M., van der Leeden M., Heymans M.W., Holla J.F., Häkkinen A., Lems W.F., Roorda L.D., Veenhof C., Sanchez-Ramirez D.C., de Vet H.C. (2016). Prognosis of pain and physical functioning in patients with knee osteoarthritis: A systematic review and meta-analysis. Arthritis Care Res..

[15] Binik Y.M., Baker A.G., Kalogeropoulos D., Devins G.M., Guttmann R.D., Hollomby D.J., Barré P.E., Hutchison T., Prud’Homme M., McMullen L. (1982). Pain, control over treatment, and compliance in dialysis and transplant patients. Kidney Int..

[16] Li X., Pan F., Zhu R., Ge L., Zhang X., Wen X., Zhou J., Cheng J., Pan F., Cai G. (2024). Cross-Sectional and Longitudinal Associations of Comorbidities with Knee Symptoms and Radiographic Abnormalities of Osteoarthritis. Rheumatol. Ther..

[17] Jinks C., Jordan K., Ong B., Croft P. (2004). A brief screening tool for knee pain in primary care (KNEST). 2. Results from a survey in the general population aged 50 and over. Rheumatology.

[18] Von Korff M., Ormel J., Keefe F.J., Dworkin S.F. (1992). Grading the severity of chronic pain. Pain.

[19] Jinks C., Lewis M., Ong B., Croft P. (2001). A brief screening tool for knee pain in primary care. 1. Validity and reliability. Rheumatology.

[20] Cavalcante R., Arruda J., Zaccariotti V., Marques R., Santos V., Fernandes Y. (2016). Evaluation between the SINS Score, VAS, and neurological status for spinal metastatic tumors. Glob. Spine J..

[21] Alghadir A., Anwer S., Iqbal Z.A., Alsanawi H.A. (2016). Cross-cultural adaptation, reliability and validity of the Arabic version of the reduced Western Ontario and McMaster Universities Osteoarthritis index in patients with knee osteoarthritis. Disabil. Rehabil..

[22] Faik A., Benbouazza K., Amine B., Maaroufi H., Bahiri R., Lazrak N., Aboukal R., Hajjaj-Hassouni N. (2008). Translation and validation of Moroccan Western Ontario and Mc Master Universities (WOMAC) osteoarthritis index in knee osteoarthritis. Rheumatol. Int..

[23] Jinks C., Jordan K., Croft P. (2002). Measuring the population impact of knee pain and disability with the Western Ontario and McMaster Universities Osteoarthritis Index (WOMAC). Pain.

[24] Terkawi A.S., Tsang S., AlKahtani G.J., Al-Mousa S.H., Al Musaed S., AlZoraigi U.S., Alasfar E.M., Doais K.S., Abdulrahman A., Altirkawi K.A. (2017). Development and validation of Arabic version of the Hospital Anxiety and Depression Scale. Saudi J. Anaesth..

[25] Mykletun A., Stordal E., Dahl A.A. (2001). Hospital Anxiety and Depression (HAD) scale: Factor structure, item analyses and internal consistency in a large population. Br. J. Psychiatry.

[26] Albatineh A.N., Al-Taiar A., Al-Sabah R., Zogheib B. (2024). Psychometric properties of the Arabic version of the Hospital Anxiety and Depression Scale in hemodialysis patients. Psychol. Health Med..

[27] Chao S., Yen M., Lin T.-C., Sung J.-M., Wang M.-C., Hung S.-Y. (2016). Psychometric properties of the kidney disease quality of life–36 questionnaire (KDQOL-36™). West. J. Nurs. Res..

[28] Schatell D., Witten B. (2008). Measuring dialysis patients’ health-related Quality of life with the KDQOL-36™. Med. Educ. Inst..

[29] Pagana K.D., Pagana T.J. (2017). Mosby’s Manual of Diagnostic and Laboratory Tests-E-Book: Mosby’s Manual of Diagnostic and Laboratory Tests-E-Book.

[30] Jhun H.-J., Sung N.-J., Kim S.Y. (2013). Knee pain and its severity in elderly Koreans: Prevalence, risk factors and impact on quality of life. J. Korean Med. Sci..

[31] Song Q., Shen P., Mao M., Sun W., Zhang C., Li L. (2020). Proprioceptive neuromuscular facilitation improves pain and descending mechanics among elderly with knee osteoarthritis. Scand. J. Med. Sci. Sports.

[32] Ganji R., Pakniat A., Armat M.R., Tabatabaeichehr M., Mortazavi H. (2018). The effect of self-management educational program on pain intensity in elderly patients with knee osteoarthritis: A randomized clinical trial. Open Access Maced. J. Med. Sci..

[33] Zempo H., Miyamoto-Mikami E., Kikuchi N., Fuku N., Miyachi M., Murakami H. (2017). Heritability estimates of muscle strength-related phenotypes: A systematic review and meta-analysis. Scand. J. Med. Sci. Sports.

[34] Glass N., Torner J., Law L.F., Wang K., Yang T., Nevitt M., Felson D., Lewis C., Segal N.A. (2013). The relationship between quadriceps muscle weakness and worsening of knee pain in the MOST cohort: A 5-year longitudinal study. Osteoarthr. Cartil..

[35] Akasbi N., Houssaini T.S., Tahiri L., Hachimi H., Maaroufi C.E., Youbi R.E., Arrayhani M., Harzy T. (2012). Rheumatic complications of long term treatment with hemodialysis. Rheumatol. Int..

[36] Hurton S., Embil J.M., Reda A., Smallwood S., Wall C., Thomson L., Zacharias J., Dascal M., Trepman E., Koulack J. (2010). Upper extremity complications in patients with chronic renal failure receiving haemodialysis. J. Ren. Care.

[37] Sulkova S., Fortova M., Valek M., Svara F. (2003). Renal bone disease. Vnitr Lek..

[38] Burston J.J., Valdes A.M., Woodhams S.G., Mapp P.I., Stocks J., Watson D.J.G., Gowler P.R.W., Xu L., Sagar D.R., Fernandes G. (2019). The impact of anxiety on chronic musculoskeletal pain and the role of astrocyte activation. Pain.

[39] Leeuw M., Goossens M.E., Linton S.J., Crombez G., Boersma K., Vlaeyen J.W. (2007). The fear-avoidance model of musculoskeletal pain: Current state of scientific evidence. J. Behav. Med..

[40] Thompson T., Keogh E., French C.C., Davis R. (2008). Anxiety sensitivity and pain: Generalisability across noxious stimuli. Pain.

[41] De Heer E.W., Ten Have M., Van Marwijk H.W., Dekker J., De Graaf R., Beekman A.T., Van Der Feltz-Cornelis C.M. (2018). Pain as a risk factor for common mental disorders. Results from the Netherlands Mental Health Survey and Incidence Study-2: A longitudinal, population-based study. Pain.

[42] Kimmel P.L., Patel S.S. (2006). Quality of life in patients with chronic kidney disease: Focus on end-stage renal disease treated with hemodialysis. Seminars in Nephrology.

[43] Davison S.N., Jhangri G.S. (2005). The impact of chronic pain on depression, sleep, and the desire to withdraw from dialysis in hemodialysis patients. J. Pain Symptom Manag..

[44] Belayev L.Y., Mor M.K., Sevick M.A., Shields A.M., Rollman B.L., Palevsky P.M., Arnold R.M., Fine M.J., Weisbord S.D. (2015). Longitudinal associations of depressive symptoms and pain with quality of life in patients receiving chronic hemodialysis. Hemodial. Int..

[45] Weisbord S.D., Mor M.K., Green J.A., Sevick M.A., Shields A.M., Zhao X., Rollman B.L., Palevsky P.M., Arnold R.M., Fine M.J. (2013). Comparison of symptom management strategies for pain, erectile dysfunction, and depression in patients receiving chronic hemodialysis: A cluster randomized effectiveness trial. Clin. J. Am. Soc. Nephrol..

[46] Kiadaliri A.A., Lamm C.J., de Verdier M.G., Engström G., Turkiewicz A., Lohmander L.S., Englund M. (2016). Association of knee pain and different definitions of knee osteoarthritis with health-related quality of life: A population-based cohort study in southern Sweden. Health Qual. Life Outcomes.

[47] Bindawas S.M., Vennu V., Auais M. (2015). Health-related quality of life in older adults with bilateral knee pain and back pain: Data from the Osteoarthritis Initiative. Rheumatol. Int..

[48] Bindawas S.M., Vennu V., Al Snih S. (2015). Differences in health-related quality of life among subjects with frequent bilateral or unilateral knee pain: Data from the Osteoarthritis Initiative study. J. Orthop. Sports Phys. Ther..

[49] Smith B.W., Zautra A.J. (2008). The effects of anxiety and depression on weekly pain in women with arthritis. Pain.

[50] Wise R.G., Lujan B.J., Schweinhardt P., Peskett G.D., Rogers R., Tracey I. (2007). The anxiolytic effects of midazolam during anticipation to pain revealed using fMRI. Magn. Reson. Imaging.

